# Evidence of overuse? Patterns of obstetric interventions during labour and birth among Australian mothers

**DOI:** 10.1186/s12884-019-2369-5

**Published:** 2019-07-04

**Authors:** Haylee Fox, Emily Callander, Daniel Lindsay, Stephanie Topp

**Affiliations:** 1 College of Public Health, Medical and Veterinary Sciences, James Cook University, Townsville, QLD 4814 Australia; 2 School of Medicine, Gold Coast Campus, Griffith University, Southport, QLD 4214 Australia

**Keywords:** Maternal Health, Obstetrics, Cesarean section, Vaginal birth, Health system

## Abstract

**Background:**

There is global concern for the overuse of obstetric interventions during labour and birth. Of particular concern is the increasing amount of mothers and babies experiencing morbidity and mortality associated with caesarean section compared to vaginal birth. In high-income settings, emerging evidence suggests that overuse of obstetric intervention is more prevalent among wealthier mothers with no medical need of it. In Australia, the rates of caesarean section and other obstetric interventions are rising. These rising rates of intervention have been mirrored by a decreasing rate of unassisted non-instrumental vaginal deliveries. In the context of rising global concern about rising caesarean section rates and the known health effects of caesarean section on mothers and children, we aim to better characterise the use of obstetric intervention in the state of Queensland, Australia by examining the characteristics of mothers receiving obstetric intervention. Identifying whether there is overuse of obstetric interventions within a population is critical to improving the quality, value and appropriateness of maternity care.

**Methods:**

The association between demographic characteristics (at birth) and birth delivery type were compared with chi-square. The percentage of mothers based on their socioeconomic characteristics were reported and differences in percentages of obstetric interventions were compared. Multivariate analysis was undertaken using multiple logistic regression to assess the likelihood of receiving obstetric intervention and having a vaginal (non-instrumental) delivery after accounting for key clinical characteristics.

**Results:**

Indigenous mothers, mothers in major cities and mothers in the wealthiest quintile all had higher percentages of all obstetric interventions and had the lowest percentages of unassisted (non-instrumental) vaginal births. These differences remained even after adjusting for other key sociodemographic and clinical characteristics.

**Conclusions:**

Differences in obstetric practice exist between economic, ethnic and geographical groups of mothers that are not attributable to medical or lifestyle risk factors. These differences may reflect health system, organisational and structural conditions and therefore, a better understanding of the non-clinical factors that influence the supply and demand of obstetric interventions is required.

## Introduction

Obstetric interventions such as caesarean section can be life-saving for mothers and newborns when medically indicated [1]. Underuse of such interventions – often stemming from lack of physical access to skilled care – has been the focus of substantial research, policy and advocacy efforts. But overuse of obstetric interventions is now an emerging global concern. The recent Lancet series ‘Optimising Caesarean Section Use’ reported a doubling in the global rate of caesarean birth in the past 15 years to 21% [1], when population rates above 10–15% are considered excessive [2, 3].

Not all attempted vaginal births are successful and the use of obstetric interventions during labour and birth in an adequately resourced health facility with appropriately trained staff can be effective for preventing perinatal morbidity and mortality [1]. For example, a caesarean section may be necessary when a vaginal delivery poses a risk to the woman or baby and when complications arise in circumstances such as fetal distress, abnormal fetal presentation, antepartum haemorrhage and hypertensive disease [4, 5]. In low-income settings where mothers have limited access to caesarean sections, data has shown an increased risk of death for both mother and baby [6]. Because of this, underuse of obstetric interventions including caesarean sections has been a major focus of literature, research, policy and funding efforts for several decades in the strive to reduce perinatal morbidity and mortality [7].

However, more recent attention has been given to the increasing evidence of *overuse* of obstetric interventions in some settings [1, 8]. Particular concerns surrounding the rising rates of caesarean section have emerged due to the increasing amount of mothers and babies experiencing morbidity and mortality associated with caesarean section compared to vaginal birth [5]. In the short term, and compared to mothers who have a vaginal birth, mothers who have a caesarean section are at higher risk of haemorrhage requiring a hysterectomy, uterine rupture, complications associated with anaesthetic, renal failure, obstetric shock, cardiac arrest, venous thromboembolism, and major puerperal infection [5, 9–11]. In the long term, mothers who have a caesarean section have an increased risk of experiencing pelvic adhesions [12], bowel obstruction [13], future subfertility [14, 15], decreased satisfaction with the birth, lower rates of breastfeeding and less positive reactions to their baby after birth [16] compared to those who have a vaginal birth. In subsequent births following caesarean section mothers are more likely to experience preterm birth and stillbirth [15, 17] and maternal death due to increased risk of uterine rupture [5].

Mothers who need obstetric care and do not receive it are faced with the risk of poor maternal health outcomes. At the same time, mothers and babies who receive unnecessary obstetric interventions are at an increased risk of iatrogenic consequences –in the short and long-term. Both the underuse and overuse of obstetric intervention – sometimes termed ‘too little too late too much too soon’ - have thus been identified as issues of global concern [18]. A related set of concerns, pertaining to the inequities implicit in the current known patterns of under and overuse. In many countries, for example, it is the poorest mothers who have inadequate access to necessary and potentially life-saving obstetric interventions [1]. While across low-, middle- and high-income settings emerging evidence suggests that overuse of obstetric intervention is more prevalent among wealthier mothers with no medical need of it [1].

In Australia, the rate of caesarean section is currently 34% (2016) and expected to continue increasing over time [19]. Concurrently, there has been a rise in the use of other obstetric interventions such as induction of labour, instrumental vaginal birth (vacuum and forceps), and episiotomy [20]. These rising rates of intervention have been mirrored by a decreasing rate of unassisted non-instrumental vaginal deliveries [20]. In the context of rising global concern about rising caesarean section rates, we aim to better characterise the use of obstetric intervention in the state of Queensland, Australia with an explicit focus on the ‘user’ side. That is, the characteristics of mothers receiving obstetric intervention.

### Background of maternal and child health in Queensland

Queensland has a population of approximately 4 million, spread across a total land area of 1,852,642 km^2^, some seven times the size of Great Britain. More than half of the state’s population lives outside the urban south-east pocket of the greater Brisbane metropolitan area, a comparatively high proportion compared to other (more urbanised) Australian states [21]. In Queensland in 2015, 60,942 mothers gave birth to 61,903 babies, which includes 3931 women who identified as Aboriginal and/or Torres Strait Islander and their 3979 babies. In Queensland, the Maternal Mortality Ratio (MMR) was 7.3 per 100,000 births and the perinatal mortality rate was 9.8 per 1000 births, this includes 6.7 stillbirths and 3.1 neonatal deaths per 1000 births (2015) [21]. These rates are not significantly different from the national figs. [19]. Whilst caesarean section is considered to contribute to the low levels of mortality, there is also the view that obstetric intervention rates are higher than desirable [22]. In Queensland in 2015, 34% of women had a caesarean section [23] and 22.5% had an instrumental birth (with either vacuum or forceps) [20].

### Access to maternal health services

Within Australia overall, reproductive health services are accessed in the first instance via a network of General Practitioners whereby mothers are advised to book in at their closest hospital that has maternity services available. The individual health service and different availability of maternal models of care determine the type of care that a mother receives during her perinatal journey [24]. In Queensland, the four main options of maternity care models include Midwifery Group Practice caseload care, private midwifery care, private obstetrician (specialist) care, and shared care, which is delivered by a combination of GP, doctors and midwives within the community and a public hospital [25]. Access to these maternity models of care depends on the services provided within each public Hospital and Health Service.

In Australia, there is a public universal healthcare system known as Medicare. Women are entitled to access their maternity care in public hospitals free of charge. However, it is also possible for women to hold private health insurance and some may choose to access care in private hospitals. Women may choose to receive private maternity care as it gives them continuity of care from a chosen obstetrician throughout pregnancy and birth. It is known, however, that women who birth privately do have higher rates of interventions [20].

### Aims

In order to better characterise the use of obstetric intervention state-wide, this study will address the following questions:

1. What are the socioeconomic characteristics of mothers who receive obstetric interventions during labour and birth?
2. What is the likelihood of having an obstetric intervention by Aboriginal and/or Torres Strait Islander status, socio-economic status and geographic status? The primary outcome of interest is a caesarean section. Secondary outcomes are instrumental vaginal birth^1^; vaginal (non-instrumental) birth^2^; the induction of labour; episiotomy; and epidural.

## Methods

### Data

This project utilised a whole of population linked dataset called Maternity1000 [26]. Maternity1000 utilises the Queensland Perinatal Data Collection (PDC) to identify all mothers who gave birth in Queensland, and currently contains the records of mothers who gave birth between 1 July 2012 and 30 June 2015 (*n* = 186,789), plus their resultant babies (*n* = 189,909).

All individuals were identified from the Queensland Perinatal Data Collection and Queensland Birth Registry by Queensland Health’s Statistical Services Branch (SSB). The records were then linked to Queensland Hospital Admitted Patient Data Collection (QHAPDC), Deaths Registry, Emergency Department Information System (EDIS) and Hospital and Health Service (HHS) Funding and Costing Unit records between 1 July 2012 and 30 June 2015. The records were then linked by the Australian Institute of Health and Welfare (AIHW) to Medicare Benefits Schedule (MBS) and Pharmaceutical Benefits Scheme (PBS) claims records [26]. However, only data from the PDC was utilised in this study.

### Index of relative socioeconomic disadvantage

Our study uses the Index of Relative Socioeconomic Disadvantage (IRSD) to categorise mothers into levels of socioeconomic position based upon their postcode of residence at the time of birth. The IRSD is compiled by the Australian Bureau of Statistics (ABS) and represents ‘the socioeconomic conditions of Australian geographic areas by measuring aspects of disadvantage’ [27]. It is designed to work like a scoring system where the attributes of populations, such as income, level of educational attainment and employment status are summarised to produce a score. Our study collapsed the IRSD decile rank into five categories (IRSD1–5). IRSD1 is the lowest socioeconomic group, which represents individuals in the study population that are living in areas with the lowest socioeconomic conditions. Conversely, IRSD5 is the highest socioeconomic group, which represents individuals the study population that are living in areas with the highest socioeconomic conditions [27].

### Mother’s rurality

To categorise mothers into levels of rurality, our study used the Accessibility/Remoteness Index of Australia (ARIA+) that was developed by the Australian Bureau of Statistics [28] to categorise women based upon their postcode of residence at the time of birth. The index scores have been classified into the following categories:

1. Major Cities — relatively unrestricted access to a wide range of goods, services and opportunities for social interaction.
2. Inner Regional — some restrictions to access to some goods, services and opportunities for social interaction.
3. Outer Regional — significantly restricted access to goods, services and opportunities for social interaction.
4. Remote — very restricted access to goods, services and opportunities for social interaction.
5. Very Remote — very little access to goods, services and opportunities for social interaction [28].

### Indigenous status

Mothers that identified at antenatal visits as either Aboriginal and/or Torres Strait Islander were recorded on the Queensland Perinatal Data Collection. In this paper, those mothers who responded ‘yes’ as either Aboriginal and/or Torres Strait Islander will be referred to as ‘Indigenous’ and those who identified as not being either Aboriginal and/or Torres Strait Islander will be referred to as ‘non-Indigenous’.

### Outcome variables

The primary outcome for this study was mothers giving birth via caesarean section. Secondary outcomes were modes of birth: instrumental vaginal birth and vaginal (non-instrumental) birth, and obstetric interventions during labour and birth: induction of labour; episiotomy; and epidural.

### Statistical analysis

The frequency and percentage of mothers who gave birth in Queensland between 1 July 2012 and 30 June 2015 were reported by IRSD, level of rurality, Indigenous status, previous pregnancy, pre-existing medical condition, plurality, and smoking status. The mean age and Body Mass Index (BMI) were also reported (Table 1). Figures on the same characteristics of mothers from the entire Australian population are also summarised in Table 1 to demonstrate the similarities and differences between the Queensland population and the Australian population. These figures were sourced from the Australian Institute of Health and Welfare (AIHW) annual *Australia’s mothers and babies* (2012–2015) reports [29–33].

**Table 1:** Maternal characteristics for mothers who gave birth in Queensland and Australia between 1 July 2012 and 30 June 2015– AIHW 2012–2015

	Queensland	Australia
Maternal characteristics	*n* = 189,811 (%)	*n* = 918,539 (%)
IRSD		
IRSD5 (least disadvantaged)	50,663 (26.99)	164,227 (18)
IRSD4	43,278 (23.05)	181,973 (20)
IRSD3	35,997 (19.18)	181,764 (20)
IRSD2	39,165 (20.86)	180,589 (20)
IRSD1 (most disadvantaged)	18,625 (9.92)	191,402 (22)
Rurality		
Major city	94,285 (50.22)	654,273 (71)
Inner regional	35,341 (18.83)	150,269 (17)
Outer regional	38,610 (20.57)	79,040 (9)
Remote	12,286 (6.54)	13,868 (2)
Very remote	7206 (3.84)	9162 (1)
Indigenous status		
No	178,133 (93.8)	879,306 (96)
Yes	11,668 (6.2)	37,849 (4)
Previous pregnancy^a^		
No	57,392 (30.24)	397215.5 (34)
Yes	132,414 (69.76)	609864.5 (66)
Pre-existing medical condition		
No	142,096 (74.86)	740,343 (81)
Yes	47,703 (25.14)	178,196 (19)
Plurality		
Singleton	183,832 (96.85)	904.634 (98)
Twins	5792 (3.05)	12,124 (1.5)
Triplets and Quadruplets	187 (0.10)	1763 (.5)
Smoking status		
No	163,337 (86.53)	744,727 (81)
Yes	25,568 (13.47)	173,813 (19)
Age (mean)	29.9	30.1
BMI (mean)	26.6	26.2

^a^Includes Livebirth, Stillbirth, Abortion (spontaneous or induced), Miscarriage, and Ectopic pregnancy

The association between demographic characteristics (at birth) and birth delivery type were compared with chi-square analyses. Due to the large sample size, we reported the Cramer’s V effect size value to determine the strengths of association between the population groups. Cramer’s V values were interpreted as per Cohen (1998)^3^ [34]. The percentage of mothers that identified as Indigenous, different levels of rurality and IRSD quintiles were reported and differences in percentages of obstetric interventions were compared. Multivariate analysis was then undertaken using multiple logistic regression to assess the likelihood of receiving obstetric intervention and having an unassisted (non-instrumental) vaginal delivery. The Odds Ratios (ORs) were calculated with adjustment for the mother having a pre-existing health condition, maternal age, previous pregnancy complications, complications arising during the current pregnancy, obesity, area-based socioeconomic deprivation, distance from the birthing facility, and smoking as potential confounding variables [35, 36]. All analysis was undertaken using SAS9.4 statistical software.

## Results

There were 189,811 babies born to mothers in Queensland between 1 July 2012 and 30 June 2015. Table 1 summarises the maternal characteristics of the mothers giving birth (both in Queensland and nationally) including Index of Relative Socioeconomic Disposition (IRSD), rurality, Indigenous status, previous pregnancy, plurality, smoking status, and the mean age and Body Mass Index (BMI). Just over one-quarter of mothers (27%) in Queensland were in the highest socio-economic quintile while 9.92 and 20.86% respectively were in the two lower quintiles. Nationally, the percentage of mothers in the middle IRSD quintiles were similar to Queensland, with the greatest differences being in the highest and lowest socio-economic quintiles (18 and 22% respectively for the national population). Six percent of Queensland mothers identified as Indigenous, which is slightly greater than the Australian population at large (4%). Matching national population profiles, almost 70% of Queensland mothers had a previous pregnancy, the average age of mothers was 30 and BMI at birth was 26.6. Queensland has a less urbanised population than the Australian population (50.22% compared to 71% living in major cities), with a greater percentage of mothers living in outer regional areas in Queensland (20.57%) compared to the national population (9%).

As seen in Table 2, Indigenous mothers had a higher percentage of unassisted vaginal births and a lower percentage of all other intervention types compared to non-Indigenous mothers. Instrumental vaginal births, episiotomies, epidurals and caesarean sections generally decreased with increasing rurality. All interventions generally increased with increasing socioeconomic status. All of the Cramer’s V effect sizes are < 0.1 and therefore indicate a small effect size.

**Table 2:** Sociodemographic characteristics of mothers receiving obstetric intervention during labour and birth in Queensland between 01/07/2012 and 30/06/2015 (percentage %)

	Caesarean section	Instrumental vaginal birth	Vaginal (non-instrumental) birth	Induction of labour	Episiotomy	Epidural
Indigenous	26.2	6.0	67.8	21.8	3.9	10.0
Non-Indigenous	34.4	10.3	55.3	24.7	6.8	16.2
*Cramer’s V*	0.0417***	0.0342***	0.0605***	0.0160***	0.0282***	0.0407***
Major city	35.7	11.3	53.1	24.6	7.7	18.0
Inner regional	32.0	9.2	58.7	24.3	5.7	16.2
Outer regional	31.8	8.7	59.5	24.3	5.7	13.2
Remote	32.7	8.1	59.3	24.1	5.7	10.5
Very remote	30.7	7.8	61.5	25.6	4.9	9.3
*Cramer’s V*	0.0404***	0.0431***	0.0640***	*0.0064*	0.0417***	0.0744***
IRSD 1	30.2	7.7	62.1	22.5	4.7	10.6
IRSD 2	32.0	8.8	59.2	24.1	5.6	14.1
IRSD 3	34.3	9.6	56.1	23.6	6.4	16.7
IRSD 4	32.2	10.1	57.6	24.9	6.1	16.2
IRSD 5	37.5	12.0	50.5	25.8	8.9	23.0
*Cramer’s V*	0.0528***	0.0470***	0.0772***	0.0245***	0.0574***	0.0611***

Significance levels are determined by Chi-square analyses. Cramer’s V values are reported due to the large sample size. *p sig at .05 **p sig at .01. ***p sig at .001

Table 3 shows the adjusted odds of obstetric intervention based on socioeconomic characteristics, after accounting for key clinical characteristics.^4^ Indigenous mothers were 0.06 times less likely to have a caesarean section than non-Indigenous mothers. Similarly, mothers in IRSD1, IRSD2 and IRSD 4 were respectively 0.07, 0.05 and 0.12 times less likely to have a caesarean section than mothers in the most wealthy IRSD5. Mothers living in inner regional areas were 0.06 times less likely to have a caesarean section than mother living in major cities.

**Table 3:** Odds ratios of obstetric interventions, adjusted for a pre-existing health condition, maternal age, previous pregnancy complications, complications arising during the current pregnancy, area-based socioeconomic deprivation, distance from the birthing facility, smoking and BMI at birth for mothers in Queensland

	Cesarean section		Instrumental vaginal birth		Vaginal (non-instrumental) birth		Induction of labour		Episiotomy		Epidural	
	OR	95%CI	OR	95%CI	OR	95%CI	OR	95%CI	OR	95%CI	OR	95%CI
Indigenous	0.94	0.90–0.99	0.70	0.65–0.77	1.14	1.09–1.19	0.86	0.82–0.90		0.65–0.80	0.74	0.69–0.79
Inner Regional	0.96	0.93–0.99	0.92	0.88–0.97	1.06	1.03–1.09	1.10	1.10–1.13	0.89	0.84–0.95	0.98	0.94–1.02
Outer Regional	1.03	1.00–1.06	0.87	0.83–0.91	1.01	0.9–1.0.4	1.10	1.07–1.14	0.94	0.89–1.00	0.79	0.76–0.82
Remote	1.10	1.05–1.15	0.77	0.72–0.83	1.00	0.96–1.04	1.05	1.00–1.10	0.88	0.80–0.95	0.57	0.54–0.61
Very remote	1.00	0.95–1.10	0.85	0.77–0.94	1.05	0.99–1.11	1.20	1.17–1.32	0.85	0.76–0.96	0.605	0.55–0.66
IRSD 2	0.93	0.89–0.97	0.80	0.75–0.86	1.15	1.10–1.20	0.79	0.75–0.83	0.63	0.58–0.69	0.72	0.68–0.77
IRSD 3	0.95	0.92–0.98	0.85	0.81–0.89	1.11	1.08–1.14	0.91	0.88–0.94	0.70	0.66–0.74	0.90	0.86–0.94
IRSD 4	0.99	0.96–1.03	0.90	0.85–0.94	1.04	1.01–1.08	0.84	0.81–0.87	0.80	0.75–0.85	0.96	0.92–1.00
IRSD 5	0.88	0.85–0.90	0.93	0.89–0.97	1.15	1.12–1.19	0.95	0.81–0.87	0.72	0.75–0.85	1.01	0.97–1.10

Indigenous mothers, mothers in inner regional areas, and mothers in all levels of socioeconomic position were all significantly more likely to have a vaginal (non-instrumental) birth than their relevant reference group (Table 3). Indigenous mothers, mothers in inner regional, outer regional and very remote regions and mothers in all socioeconomic quintiles were less likely than their relevant reference group to have their labour induced.

Indigenous mothers and mothers from all levels of socioeconomic position were all less likely to have an instrumental vaginal delivery. Indigenous mothers, mothers in inner regional and very remote areas, and mothers from all levels of socioeconomic position were less likely to have an instrumental vaginal delivery. Indigenous mothers and mothers from all levels of rurality and all socioeconomic quintiles were less likely to have an epidural.

## Discussion

The aim of this study was to examine the likelihood of receiving obstetric interventions during labour and birth based on Indigenous, socio-economic and geographic status. Non-Indigenous mothers, mothers in major cities and mothers in the wealthiest quintile all had higher percentages of all obstetric interventions and had the lowest percentages of vaginal (non-instrumental) births. These differences remained even after adjusting for other key socio-economic, demographic and clinical characteristics.

Prima facie, we would expect the percentage of obstetric interventions to be higher among population groups with known higher rates of maternal risk factors. In Australia, Indigenous mothers, mothers from socioeconomically disadvantaged backgrounds and mothers residing in rural and remote regions attend fewer antenatal appointments [19], experience higher rates of smoking during pregnancy [20], are more likely to be obese and have a higher prevalence of pre-existing medical conditions such as hypertension, diabetes and gestational diabetes [19]. These factors are all associated with increased risk during pregnancy and birth and increase the potential need for obstetric intervention [19, 37–40].

Our adjusted figures, however, demonstrate the pattern of intervention in Queensland to be the inverse of these expectations with mothers in the wealthiest quintile having significantly higher odds of having a caesarean section, induction, episiotomy, epidural and instrumental vaginal birth than mothers in the poorest quintile. Even after adjusting for known clinical risk factors, the likelihood that wealthier, non-Indigenous, urban-based mothers received obstetric intervention remained significant. Given the relatively high intervention rates in Australia, these results are strongly suggestive of a pattern of overuse, as has been demonstrated in other countries [1].

The analysis presented here does not enable us to assess whether mothers from geographically, economically or ethnically marginalised groups are receiving too few interventions. However, evidence from the literature suggests that caesarean section levels above 10–15% do not reduce maternal or perinatal mortality rates within a population [2, 3], with all groups of mothers reported in our study having caesarean section rates well above this. Our results do raise the urgent question as to why wealthier, urban, and non-Indigenous mothers are receiving such relatively high rates of obstetric intervention, even after adjusting for medical and lifestyle risk factors?

Variation in obstetric intervention rates has been reported globally between geographic regions, between wealth quintiles [1, 41] between states and territories within countries [20], between public and private hospitals [42], between different ethnic groups [43], and between hospitals [44]. Boatin et al. [45] examined differences in caesarean section rates between wealth quintiles in 72 low and middle income countries, which found that overall, caesarean section rates were lower amongst the poorest wealth quintile and higher among the richest wealth quintile, with only three European countries having higher caesarean section rates in the poorest fifth than the richest fifth. The results of their study are comparable to ours, which found significantly higher caesarean section rates among the least disadvantaged quintile compared to the most disadvantaged quintile.

Little systematic work has been done to capture Australian mothers’ preferences regarding birth and obstetric intervention, although one study conducted in 2007 suggests that few mothers want a caesarean section in the absence of a clinical need for it [46]. Some pregnant mothers may choose, or agree to birth via Caesarean section due to non-medical factors. Fear of birth [47–51], previous birth experience [47, 51], concerns about the safety of a vaginal delivery [52], health provider influence [46, 47, 53, 54], misinformation [47, 53], and social norms and expectations [55, 56] may all play a part in the decision to have a caesarean delivery. In Australia, 24% of pregnant mothers experience fear of birth [57], with multiple Australian studies [49, 50, 58] reporting a greater likelihood of having a caesarean section for mothers who experience fear of childbirth during pregnancy. Consideration should also be given to the influence that care providers may have on a woman’s decision to have a caesarean section [59, 60]. Currently, there is a lack of research that reports on the interactions between women and their care providers and the information provided to women when they choose to have a caesarean birth. One Australian study [38] that surveyed pregnant women on their recollection of discussions with health providers on the risks and benefits of caesarean section for themselves and their baby reported that women who preferred to have a caesarean section were typically poorly informed about the associated risks for themselves and their baby.

The clinical outcome data that was utilised in this study is routinely collected across Australia [26]. The strength of this data source is the ability to generate results for an entire population, as opposed to a selected sample, and also the completeness of Indigenous status identification. This reduces the potential for sampling bias, as it does not limit the sample size of mothers from minority population groups that are often underrepresented in healthcare research [61]. However, the limitations of this study are the measure of socioeconomic disadvantage is area based and not measured at the individual level. Additionally, clinical outcomes are not woman-centered, which means that such measures do not directly capture whether the outcomes of importance to birthing women are met.

## Conclusion

This study has demonstrated that differences in obstetric practice exist between economic, ethnic and geographical groups of mothers that are not attributable to medical or lifestyle risk factors in Queensland, Australia. Rather, differences may reflect health system, organisational and structural conditions. To deliver maternity care that is equitable and of high quality, there needs to be a systems thinking approach to better understand the non-clinical factors that influence the supply and demand of obstetric interventions. Serious consideration at the government, organisational and health provider level of how to reduce the potentially inappropriate use of obstetric interventions and the consequential iatrogenic conditions that can result from unwarranted use is essential.

### Aknowledgements

Not applicable.

## Data Availability

Data sharing is not available for this study due to privacy considerations. Ethical approval for this study explicitly prohibits the sharing of our dataset.

## Abbreviations

ABS: Australian Bureau of Statistics
AIHW: Australian Institute of Health and Welfare
ARIA: Accessibility/Remoteness Index of Australia
BMI: Body Mass Index
EDIS: Emergency Department Information System
GP: General Practitioner
HHS: Hospital and Health Service
IRSD: Index of Relative Socio Economic Disadvantage
MBS: Medicare Benefits Schedule
OR: Odds Ratio
PBS: Pharmaceutical Benefits Scheme
PDC: Perinatal Data Collection
QHAPDC: Queensland Health Admitted Patient Data Collection

## Footnotes

1 Instrumental vaginal birth is a medically assisted birth with the use of obstetrical instruments – either a vacuum cup or forceps.

2 A birth which is achieved solely by the mother’s expulsive efforts requiring no mechanical or surgical assistance.

3 Cramer’s V varies between 0 and 1 with < 0.1 denoting a small effect size, 0.1–0.29 a medium effect size and 0.3–0.5 a large effect size.

4 The reference group for Indigenous is non-Indigenous, for rurality it is major city and for IRSD it is IRSD 1.
